# Effect of the phosphodiesterase 4 inhibitor apremilast on cardiometabolic outcomes in psoriatic disease—results of the Immune Metabolic Associations in Psoriatic Arthritis study

**DOI:** 10.1093/rheumatology/keab474

**Published:** 2021-06-07

**Authors:** Lyn D Ferguson, Susanne Cathcart, Dominic Rimmer, Gary Semple, Katriona Brooksbank, Caron Paterson, Rosemary Brown, John Harvie, Xuan Gao, Aleksandra Radjenovic, Paul Welsh, Iain B McInnes, Naveed Sattar, Stefan Siebert

**Affiliations:** 1 Institute of Cardiovascular and Medical Sciences, University of Glasgow; 2 Glasgow Clinical Research Facility, Glasgow Royal Infirmary; 3 Institute of Infection, Immunity, and Inflammation, University of Glasgow, Glasgow; 4 Raigmore Hospital, Inverness, UK

**Keywords:** apremilast, PDE4 inhibition, weight, metabolic, vascular, adipose tissue, ectopic fat, psoriatic disease

## Abstract

**Objectives:**

Studies have suggested phosphodiesterase 4 (PDE4) inhibition may be associated with weight loss and other cardiometabolic benefits. We evaluated the effect of the PDE4 inhibitor apremilast on body weight and composition, glucose homeostasis, lipid profiles and vascular function in psoriatic disease and whether weight change correlated with therapeutic response.

**Methods:**

We conducted a prospective, open-label study (Immune Metabolic Associations in Psoriatic Arthritis) of adults receiving apremilast 30 mg as part of routine care for PsA and/or psoriasis. Cardiometabolic, anthropometric and disease activity assessments were performed at baseline (pre-apremilast) and at months 1, 3 and 6 of apremilast treatment in 60 patients. A subgroup underwent further assessment of endothelial function, body composition and adipocyte morphology.

**Results:**

In patients (median age 54.5 years, 63% women, median BMI 33.2 kg/m^2^), apremilast was associated with a mean weight loss of 2.2 kg (95% CI 1.4, 3.0; *P* < 0.001) and a mean BMI decrease of 0.8 kg/m^2^ (95% CI 0.5, 1.2; *P* < 0.001) after 6 months of treatment. Body composition analysis demonstrated a reduction in total abdominal fat [mean decrease 0.52 L (95% CI 0.08, 0.96), *P* = 0.022], principally subcutaneous adipose tissue [mean decrease 0.37 L (95% CI 0.05, 0.68), *P* = 0.022]. There was no change in adipocyte diameter, haemoglobin A1c, lipid, glucagon-like peptide-1 or vascular function. Psoriatic disease activity improved with apremilast, although this was not correlated with weight change.

**Conclusion:**

Following apremilast treatment, we observed weight loss, principally abdominal subcutaneous fat, and improvement in psoriatic disease activity. The latter was independent of weight change, suggesting apremilast likely acts through direct immunological mechanisms.

Rheumatology key messages

- Apremilast was associated with weight loss, principally abdominal subcutaneous fat.
- There was no change in HbA1c, GLP-1, lipid or vascular function parameters with apremilast.
- Improvements in disease activity were independent of weight change, suggesting apremilast likely acts through immunological rather than metabolic effects.

## Introduction

Psoriasis and PsA are associated with significant cardiometabolic comorbidities including obesity, type 2 diabetes and cardiovascular disease [1]. Excess adipose tissue is thought to contribute to chronic low-grade inflammation and may play an important role in psoriatic disease pathogenesis and treatment response. Recent studies have shown that >5% weight loss is associated with a higher rate of achievement of minimal disease activity in individuals with PsA and obesity [2, 3].

The management of psoriatic disease has focussed mainly on anti-inflammatory and immune-modulating therapies. One such therapy, apremilast, reduces inflammation through inhibiting the small molecule phosphodiesterase 4 (PDE4) and increasing intracellular cyclic adenosine monophosphate [4]. In addition to improvements in psoriatic disease activity, apremilast has been reported to be associated with weight loss [5, 6] and, in post-hoc analysis, with modest reductions in haemoglobin A1c (HbA1c) [7]. It has been previously suggested these effects may be mediated by enhanced glucagon-like peptide-1 (GLP-1) activity [8]. A preliminary study has also suggested potential vascular benefits of PDE4 inhibition, with improved endothelial function in patients treated with apremilast, although these findings were limited by small sample size [9].

Collectively this suggests PDE4 inhibition may have additional cardiometabolic effects beyond its anti-inflammatory role in psoriatic disease. Such improvements in metabolic outcomes may contribute to improved disease activity as well as associated comorbidities.

This study aimed to characterize dynamic changes in cardiometabolic profiles in individuals with psoriatic disease treated with apremilast and whether weight change correlated with therapeutic response. Primary objectives included assessing the change in body weight and glucose homeostasis with PDE4 inhibition. Secondary objectives included evaluating the nature of PDE4 inhibitor–associated weight loss through MRI analysis of body composition and abdominal adipocyte morphology, as well as assessing a potential mechanism for glycaemic modulation by apremilast through evaluation of pre- and post-prandial GLP-1 levels. Modulation of lipid profiles and vascular parameters including blood pressure, endothelial function and arterial stiffness with apremilast treatment were also assessed.

## Patients and methods

### Study design and participants

The Immune Metabolic Associations in Psoriatic Arthritis (IMAPA; ClinicalTrials.gov NCT03399708) study was a prospective open-label study that recruited adult patients with PsA and/or psoriasis registered at rheumatology/dermatology clinics in NHS Greater Glasgow and Clyde, NHS Lanarkshire and NHS Highland between June 2017 and May 2019 who were starting apremilast as part of standard clinical care. Participants had to fulfil the Classification for Psoriatic Arthritis criteria for PsA [10] or have chronic plaque psoriasis confirmed by a dermatologist.

Exclusion criteria included immune-mediated rheumatic disease other than PsA or psoriasis; severe renal disease (estimated glomerular filtration rate ≤30 ml/min); liver disease with alanine aminotransferase or aspartate aminotransferase >4 times the upper limit of normal; haemoglobin ≤9 g/dl; inflammatory bowel disease or coeliac disease; cancer currently receiving chemo- or radiotherapy; severe depression and/or history of suicidal ideation; current use of leflunomide or biologics; oral or i.m. steroids within 6 weeks of baseline; clinically meaningful weight loss >3 kg, current/planned use of weight loss medication or severe calorie restriction within the first 3 months of the study; current insulin therapy, GLP-1 agonist or dipeptidyl peptidase-4 (DPP-IV) inhibitor use; statin therapy started/stopped or dose altered within 3 months of the baseline visit; thyroxine started or dose altered within 6 weeks of baseline; acitretin within 8 weeks of baseline and pregnancy or breastfeeding.

The decision to start apremilast was made independently of the study by the patient’s usual rheumatology/dermatology team and was prescribed as part of routine clinical care, with standard dose titration to a maintenance dose of 30 mg twice daily or, if unable to tolerate a full dose, 30 mg once daily. All participants were included in the main study, consisting of four visits: baseline (pre-apremilast) and months 1, 3 and 6 of apremilast treatment, where cardiometabolic, anthropometric and psoriatic disease activity assessments were performed. A nested cohort of participants also entered the substudy, which included more detailed cardiometabolic phenotyping with assessment of endothelial function by EndoPAT (Itamar Medical, Caesarea, Israel), body composition analysis by whole-body MRI and subcutaneous adipose tissue biopsy.

All patients in the study provided written informed consent. The study was approved by the West of Scotland Regional Ethics Service Research Ethics Committee 4 (reference 17/WS/0006).

### Outcomes

Body weight (kg) and height (m) were measured at each visit by trained research nurses with the participant in light clothes and without shoes. BMI was calculated as weight (kg)/height (m2). Waist circumference (cm) was measured at the midpoint between the lower margin of the last palpable rib and the top of the iliac crest. Hip circumference (cm) was measured around the widest portion of the buttocks with the participant’s heels together. Waist:hip ratio was calculated as the waist circumference divided by the hip circumference. Blood pressure was measured after the participant was seated for 5 min.

Participants were asked to fast overnight before attending their morning study visit where blood samples were taken for fasting glucose, insulin, GLP-1, HbA1c and lipid profiles. At the baseline, month 3 and month 6 visits an oral glucose tolerance test (OGTT) was undertaken with serial measurements of plasma glucose, insulin and GLP-1 at 30 min intervals for a total of 2 h. The homeostasis model assessment (HOMA) 2 calculator was used to calculate insulin resistance (HOMA-IR), beta cell function and insulin sensitivity [11].

Plasma glucose was analysed on the Roche/Hitachi cobas c311 system (Roche Diagnostics, Rotkreuz, Switzerland) by the enzymatic method with hexokinase and ultraviolet detection. Plasma insulin was measured using an electrochemiluminescence immunoassay (ECLIA) technique (Elecsys Insulin assay, Roche). Whole-blood HbA1c was analysed on the cobas c311 system (Roche) based on the turbidimetric inhibition immunoassay. Serum total cholesterol, high-density lipoprotein cholesterol (HDL-C) and triglycerides were measured on the cobas c311 system (Roche) by an enzymatic colorimetric method. Low-density lipoprotein cholesterol (LDL-C) was calculated using the Friedewald equation (all concentrations expressed in mmol/l) [12]: LDL-C = Total cholesterol −HDL-C − (Triglycerides/2.22). Total GLP-1 was measured using the Northern Lights Mercodia Total GLP-1 NL-ELISA (Mercodia, Uppsala, Sweden).

Disease activity was assessed at each visit by trained research nurses. This included examination of the 66 swollen/68 tender joint counts and 28-joint DAS with ESR (DAS28-ESR), Leeds enthesitis index, dactylitis count, Psoriasis Area Severity Index (PASI), patient pain visual analogue scale (VAS), patient global assessment VAS, physician global assessment VAS and HAQ Disability Index. The minimal disease activity (MDA) score was calculated from the constituent scores [13]. Blood was also collected at each visit to measure CRP and ESR.

An optional substudy was offered to local participants with PsA at one site (Glasgow), where more detailed cardiometabolic phenotyping was assessed at baseline and after 3 months of apremilast treatment. This included whole-body MRI with assessment of abdominal body fat and lean tissue distribution using a 3.0 T Prisma magnetic resonance scanner with a dual-echo Dixon Vibe protocol (Siemens, Munich, Germany). Body composition parameters were derived as previously described [14] and included abdominal subcutaneous adipose tissue (ASAT) volume, ASAT index (ASAT normalized by height squared), visceral adipose tissue (VAT) volume, VAT index (VAT normalized by height squared), total abdominal fat (TAF) volume, TAF index (TAF normalized by height squared), liver fat percentage, muscle fat infiltration, total thigh fat-free muscle volume and weight:muscle ratio.

Remaining substudy components included abdominal subcutaneous adipose tissue biopsy. Between 100 and 300 mg of superficial abdominal subcutaneous adipose tissue was aspirated and analysed to assess adipocyte diameter and distribution (percentage of small, medium and large adipocytes). Endothelial function was assessed using the EndoPAT 2000 device (Itamar Medical). This non-invasive device employs peripheral arterial tonometry technology to measure peripheral microvascular endothelial function by assessing changes in digital pulse volume in the fingertips during a period of reactive hyperaemia [15]. A reactive hyperaemia index was calculated by the EndoPAT software and a value ≤1.67 is suggestive of endothelial dysfunction [16]. The peripheral augmentation index, a measure of arterial stiffness corrected to a heart rate of 75 bpm, was also automatically calculated from pulse wave analysis of the signal measured by the EndoPAT device.

### Statistical analysis

All analyses were performed using Stata version 14 (StataCorp, College Station, TX, USA). Continuous data were presented as mean and s.d. and categorical data as number and percentage. Continuous variables were checked for normality by visual inspection of histograms. Data were compared pre- and post-apremilast at baseline and 1, 3 and 6 months of treatment. Changes in primary and secondary outcomes in response to PDE4 inhibition were analysed using repeated measures mixed models. The total area under the curve (AUC) for glucose, insulin and GLP-1 response during the OGTT was calculated in Stata version 14 (StataCorp) using the pksumm and pkcollapse functions. Correlation between weight change and disease activity outcomes and weight change with fasting glycaemic and insulin parameters were assessed by Pearson correlation. Fisher’s exact test was used to compare weight change with achievement of MDA. *P*-values <0.05 were considered statistically significant.

## Results

### Study population characteristics

**Fig. 1:**
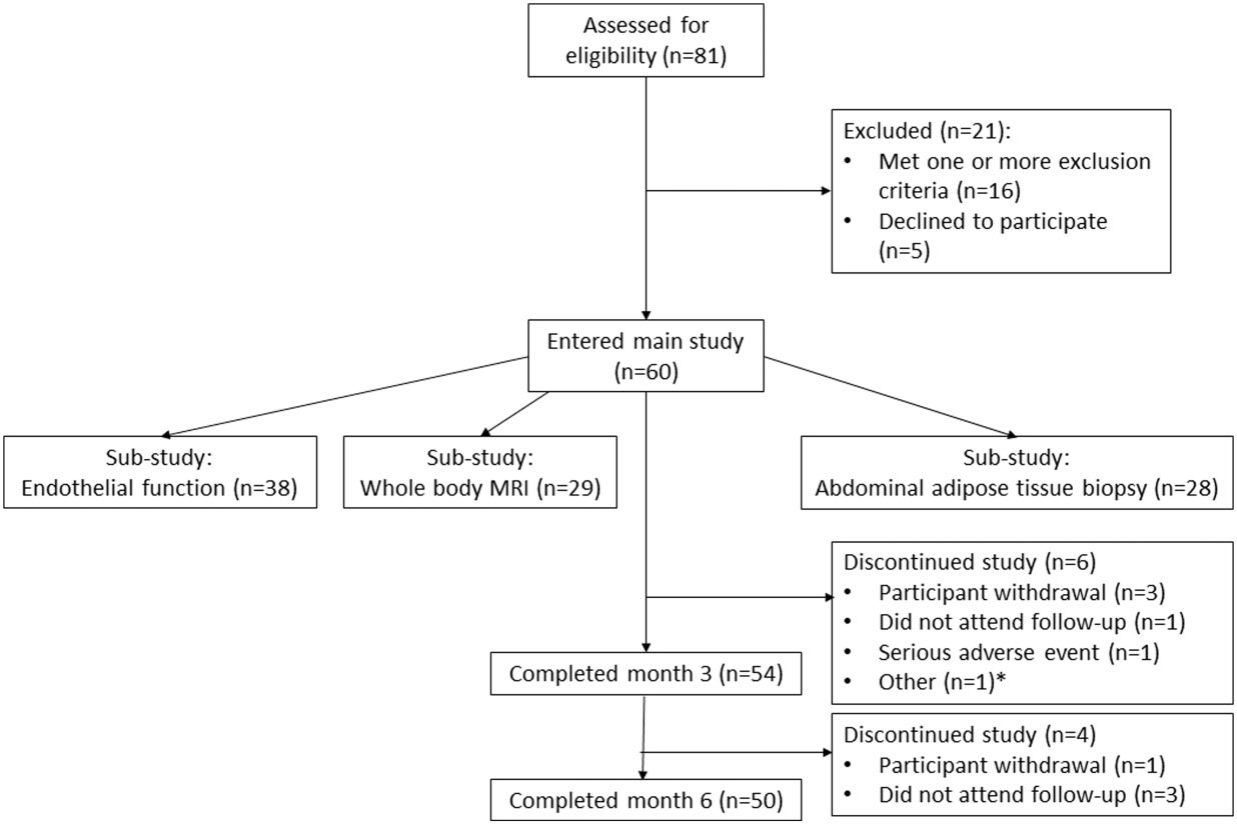
IMAPA study enrolment diagram *One participant had inadequate venous access.

A total of 60 patients were enrolled in the main study (56 patients were prescribed apremilast for PsA and 4 for psoriasis); 54 (90%) completed to month 3 and 50 (83.3%) completed to the last study visit (month 6) [4 patients withdrew from the study, 4 did not attend follow-up, 1 had a serious adverse event (anxiety episode with suicidal ideation) and 1 had inadequate venous access] (Fig. 1). In the substudies, 38 participants underwent endothelial function assessment, 29 underwent whole-body MRI and 28 had abdominal adipose tissue biopsies. Participant baseline characteristics are shown in Table 1.

**Table 1:** Participant baseline characteristics

Characteristics	Values
Age (years), median (IQR)	54.5 (42.5–62)
Sex, female/male, *n* (%)	38 (63)/22 (37)
Indication for apremilast, *n* (%)	
PsA	56 (93)
Psoriasis	4 (7)
Disease duration, years, median (IQR)	
PsA	7.8 (1.8–11.9)
Psoriasis	14.7 (6.8–23.3)
Smoking status, *n* (%)	
Current smoker	13 (22)
Ex-smoker	15 (25)
Non-smoker	31 (52)
Not stated	1 (2)
Anthropometry, median (IQR)	
Weight, kg	93.0 (75.8–107.2)
BMI, kg/m^2^	33.2 (26.8–40.0)
Waist circumference, cm	103.5 (95–120)
Hip circumference, cm	113 (102.5–127)
Waist:hip ratio	0.93 (0.88–0.98)
Systolic BP (mmHg)	132 (120–146)
Diastolic BP (mmHg)	78 (70–84)
Comorbidities, *n* (%)	
Hypertension	13 (21.7)
Type 2 diabetes	4 (6.7)
Dyslipidaemia	6 (10.0)
Liver disease^a^	4 (6.7)
Medications, *n* (%)	
Anti-hypertensive agents	14 (23.3)
Lipid-lowering therapy	8 (13.3)
Oral anti-diabetic agents^b^	1 (1.7)
Concomitant DMARD	11 (18.3)

a Liver disease consisted of *n* = 2 non-alcoholic fatty liver disease, *n* = 1 alcoholic liver disease and *n* = 1 liver fibrosis and cyst. ^b^Excluding DPP-IV inhibitors.

### Psoriatic disease activity

There were improvements in tender and swollen joint counts, DAS28-ESR score, physician and patient assessed global activity, pain VAS, Leeds enthesitis index and PASI scores after 6 months of apremilast treatment (Supplementary Table S1, available at *Rheumatology* online). Of the 49 participants with PsA who completed to month 6 and had relevant data available, 8 (16.3%) achieved MDA.

### Adiposity

**Fig. 2:**
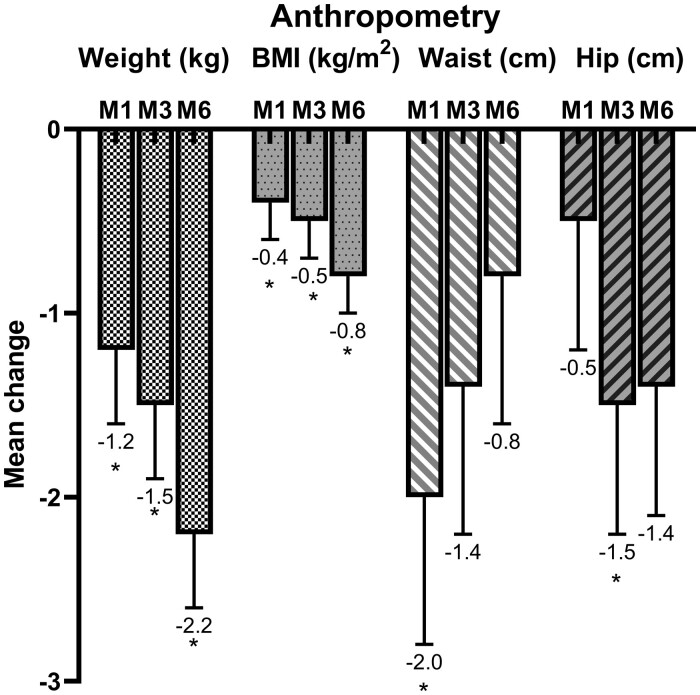
Mean changes in weight, BMI and waist and hip circumferences from baseline to months 1 (M1), 3 (M3) and 6 (M6) of treatment Error bars represent standard error (SE). *P < 0.05.

There were reductions in weight and BMI with apremilast treatment across all time points compared with baseline, with a mean weight loss of 2.2 kg (95% CI 1.4, 3.0; *P* < 0.001) and a mean BMI decrease of 0.8 kg/m^2^ (95% CI 0.5, 1.2; *P* < 0.001) by the end of the study. While waist circumference decreased significantly at month 1 and hip circumference at month 3, there was no overall change in waist:hip ratio at the end of the study (Fig. 2). A total of 7.6% (4/53) and 20.4% (10/49) of participants lost >5% body weight after 3 and 6 months of treatment, respectively.

When assessing the nature of weight loss with apremilast on MRI, there were reductions in total abdominal fat volume [mean decrease 0.52 L (95% CI 0.08, 0.96), *P* = 0.022], including ASAT volume [mean decrease 0.37 L (95% CI 0.05, 0.68), *P* = 0.022]. There were no significant changes in ectopic fat compartments, including VAT, liver fat fraction or muscle fat infiltration, nor in fat-free muscle volume (Table 2). There was no significant change in mean adipocyte diameter or the distribution of small, medium and large adipocytes with apremilast treatment (Table 3).

**Table 2:** Body composition parameters at baseline and change with 3 months of apremilast treatment

Parameters	Baseline, median (IQR)	Change at month 3, mean (95% CI)	*P*-value
VAT, l	5.76 (4.51–6.58)	−0.15 (−0.34, 0.03)	0.102
VAT index, l/m^2^	1.99 (1.43–2.39)	−0.05 (−0.11, 0.02)	0.151
ASAT, l	9.05 (6.13–15.32)	−0.37 (−0.68, −0.05)	**0.022**
ASAT index, l/m^2^	2.97 (1.87–4.67)	−0.13 (−0.25, −0.01)	**0.040**
Total abdominal fat, l	14.24 (12.64–20.14)	−0.52 (−0.96, −0.08)	**0.022**
Total abdominal fat index, l/m^2^	5.56 (4.12–7.87)	−0.17 (−0.34, −0.01)	**0.043**
Liver fat, %	8.12 (4.00–13.89)	−0.18 (−1.91, 1.55)	0.839
Muscle fat infiltration, %	7.34 (5.53–9.04)	0.01 (−0.16, 0.18)	0.950
Thigh fat-free muscle volume, l	10.57 (8.57–12.05)	−0.11 (−0.22, 0.00)	0.055
Weight:muscle ratio, kg/l	8.24 (7.22–10.77)	−0.04 (−0.09, 0.02)	0.201

*N* = 29. The values in bold are statistically significant, i.e. *P* < 0.05.

**Table 3:** Adipocyte diameter and distribution of small, medium and large adipocytes at baseline and change with 3 months of apremilast treatment

Adipocyte characteristics	Baseline, mean (s.e.)	Change at month 3, mean (95% CI)	*P*-value
Diameter, µm	96.1 (2.1)	2.6 (−2.0, 7.2)	0.262
Cell size distribution, %			
Small (≤50 µm)	7.6 (0.9)	−0.7 (−3.5, 2.1)	0.615
Medium (>50–≤100 µm)	46.5 (4.5)	−5.4 (−13.8, 3.1)	0.212
Large (>100 µm)	45.9 (4.4)	6.2 (−2.6, 15.0)	0.170

*N* = 28.

Aside from a negative correlation between weight change and patient global activity, there was no correlation between weight change and remaining disease activity parameters (Supplementary Table S2, available at *Rheumatology* online). There was no significant difference in achievement of MDA in those who lost weight compared with those who gained weight after 6 months treatment (MDA achievement 17.6% *vs* 14.3%, respectively; *P* = 1.00).

### Glycaemic, insulin & GLP-1 parameters

There were no significant changes in HbA1c, post-prandial glucose or post-prandial insulin parameters over the 6 month treatment period (Table 4). A small increase in fasting glucose [mean change 0.5 mmol/l (95% CI 0.1, 0.9), *P* = 0.012] was observed after 6 months of therapy. Fasting insulin and HOMA-IR increased with apremilast treatment, with a corresponding decrease in insulin sensitivity and an increase in beta cell function (Table 4). There were no significant changes in fasting, 2 h or total AUC for GLP-1 concentrations with apremilast treatment. There was no correlation between the change in fasting glucose, fasting insulin or HOMA-IR with weight change (Supplementary Table S3, available at *Rheumatology* online).

**Table 4:** Glycaemic, insulin, GLP-1 and lipid parameters at baseline and change with treatment

Variable	Baseline (*n* = 59)	Change at month 1 (*n* = 55)	*P*-value	Change at month 3 (*n* = 53)	*P*-value	Change at month 6 (*n* = 50)	*P*-value
HbA1c (mmol/mol)	38.0 (7.1)	−0.9 (−2.2, 0.4)	0.185	0.2 (−1.1, 1.6)	0.748	1.1 (−0.2, 2.5)	0.105
OGTT glucose							
Fasting glucose (mmol/L)	5.3 (4.9–5.8)	0.0 (−0.4, 0.4)	0.912	0.1 (−0.3, 0.5)	0.582	0.5 (0.1, 0.9)	**0.012**
30 min glucose (mmol/L)	9.3 (8.2–10.8)	–	–	−0.1 (−0.7, 0.5)	0.805	0.2 (−0.5, 0.8)	0.577
1 h glucose (mmol/L)	9.1 (7.0–12.3)	–	–	0.0 (−0.7, 0.7)	0.971	0.2 (−0.6, 0.9)	0.632
90 min glucose (mmol/L)	7.5 (6.5–10.7)	–	–	−0.1 (−0.8, 0.6)	0.826	−0.3 (−1.0, 0.4)	0.429
2 h glucose (mmol/L)	6.9 (5.8–8.3)	–	–	−0.5 (−1.1, 0.2)	0.175	−0.3 (−1.0, 0.4)	0.341
Glucose AUC (mmol/min/L)	979 (847–1215)	–	–	−11 (−78, 57)	0.755	8 (−63, 79)	0.829
OGTT insulin							
Fasting insulin (µU/ml)	13.4 (9.1–20.1)	4.7 (1.2, 8.2)	**0.008**	3.6 (0.1, 7.1)	**0.046**	5.4 (1.9, 9.0)	**0.003**
30 min insulin (µU/ml)	76.0 (49.6–128.7)	–	–	−0.9 (−23.0, 21.3)	0.940	0.9 (−22.1, 23.9)	0.939
1 h insulin (µU/ml)	100.2 (59.5–146.9)	–	–	10.6 (−11.6, 32.9)	0.349	−4.8 (−28.1, 18.4)	0.683
90 min insulin (µU/ml)	95.4 (52.1–144.8)	–	–	8.8 (−9.4, 27.1)	0.344	−11.7 (−30.5, 7.1)	0.224
2 h insulin (µU/ml)	65.7 (42.9–116.7)	–	–	−8.2 (−26.7, 10.3)	0.384	−15.0 (−34.3, 4.4)	0.129
Insulin AUC (µU/min/mL)	10209 (6549–13 600)	–	–	422 (−1411, 2255)	0.652	−554 (−2477, 1369)	0.572
OGTT GLP-1							
Fasting GLP-1 (pmol/L)	5.81 (4.25–7.58)	0.94 (−0.09, 1.97)	0.073	0.94 (−0.10, 1.99)	0.077	0.88 (−0.19, 1.94)	0.106
30 min GLP-1 (pmol/L)	13.49 (10.22–20.37)	–	–	−2.87 (−8.93, 3.19)	0.353	−0.26 (−6.59, 6.07)	0.936
1 h GLP-1 (pmol/L)	10.20 (7.16–17.46)	–	–	−3.44 (−6.37, −0.51)	**0.021**	−1.51 (−4.57, 1.55)	0.334
90 min GLP-1 (pmol/L)	8.21 (5.04–12.60)	–	–	−0.80 (−3.64, 2.05)	0.584	−0.92 (−3.85, 2.00)	0.536
2 h GLP-1 (pmol/L)	5.87 (4.40–9.94)	–	–	−1.53 (−3.49, 0.44)	0.128	−0.56 (−2.61, 1.50)	0.596
GLP-1 AUC (pmol/min/L)	41.21 (28.82–58.60)	–	–	−7.75 (−20.62, 5.12)	0.238	−2.75 (−16.19, 10.69)	0.689
HOMA indices							
HOMA-IR	2.0 (1.3–3.0)	0.65 (0.19, 1.11)	**0.006**	0.50 (0.03, 0.96)	**0.037**	0.75 (0.27, 1.23)	**0.002**
HOMA-%S	50.1 (33.4–77.5)	−10.4 (−16.2, −4.6)	**<0.001**	−6.6 (−12.5, −0.7)	**0.029**	−8.7 (−14.7, −2.6)	**0.005**
HOMA-%β	122 (102.3–168)	17.3 (4.6, 30.0)	**0.008**	14.6 (1.7, 27.5)	**0.026**	10.0 (−3.2, 23.3)	0.137
Lipid profiles							
Total cholesterol (mmol/L)	4.7 (1.0)	−0.2 (−0.4, −0.0)	**0.043**	−0.2 (−0.4, 0.0)	0.086	0.1 (−0.1, 0.3)	0.451
HDL-C (mmol/L)	1.3 (1.1–1.6)	−0.1 (−0.1, −0.0)	**0.040**	−0.1 (−0.1, −0.0)	**0.030**	−0.0 (−0.1, 0.1)	0.912
LDL-C (mmol/L)	2.7 (0.9)	−0.1 (−0.3, 0.0)	0.092	−0.1 (−0.3, 0.0)	0.067	0.0 (−0.2, 0.2)	0.952
Triglycerides (mmol/L)	1.2 (0.8–1.8)	−0.0 (−0.3, 0.2)	0.922	0.1 (−0.2, 0.3)	0.444	0.2 (−0.1, 0.4)	0.178

Baseline values are mean (s.d.) (normally distributed) or median (IQR) (not normally distributed variables); mean change (95% CI) compared with baseline. Values in bold are statistically significant, i.e. *P* < 0.05. Baseline data available for n = 59. HOMA: homeostasis model assessment; HOMA-IR: homeostasis model assessment of insulin resistance; HOMA-%β: beta-cell function; HOMA-%S: insulin sensitivity.

### Lipid parameters

There were initial modest reductions in total cholesterol, HDL-C and LDL-C concentrations with treatment, however, these returned to within baseline after 6 months. There was no significant change in triglycerides concentration with treatment (Table 4).

### Vascular parameters

While systolic blood pressure appeared to decrease after 6 months of apremilast treatment [mean change −4.1 mmHg (95% CI −8.1, −0.1), *P* = 0.042], this was no longer significant after exclusion of two participants commencing antihypertensive medication during the study [mean change −2.8 mmHg (95% CI −6.7, 1.1), *P* = 0.163]. While diastolic blood pressure appeared to initially increase, there was no change by the end of the study at 6 months (Supplementary Table S4, available at *Rheumatology* online).

The median reactive hyperaemia index at baseline was 2.21 [interquartile range (IQR) 1.82–2.70] greater than the manufacturer’s recommended cut-off of 1.67 and not suggestive of endothelial dysfunction; there was no statistically significant change with apremilast treatment. The baseline median augmentation index was 9.14% (IQR 1.51–19.6), with no significant change after treatment (Supplementary Table S4, available at *Rheumatology* online).

## Discussion

Following apremilast treatment, we observed weight loss, principally of abdominal subcutaneous fat, and improvement in psoriatic disease activity. Improvement in disease activity appeared independent of weight change. Our study is in keeping with a previous post-hoc analysis of the Efficacy and Safety Trial Evaluating the Effects of Apremilast in Psoriasis 1 and 2 (ESTEEM 1 and ESTEEM 2) phase 3 trials that revealed a mean weight loss of 1.51 kg (s.d. 3.85) after 16 weeks of apremilast treatment (compared with 0.01 kg with placebo) [17]. Given that we have recently shown individuals with PsA have a metabolically adverse body composition with greater visceral and ectopic liver fat, which is in turn associated with increased cardiometabolic disease propensity [14], we wished to ascertain whether weight loss with apremilast altered these regions. While there was a reduction in abdominal subcutaneous fat with apremilast, we did not observe any significant reductions in visceral or liver fat. These latter findings may be limited by the small sample size and relatively short duration of the substudy.

In addition to the site of weight loss, assessment of adipocyte morphology can provide useful information on associated metabolic comorbidities. Enlarged subcutaneous abdominal adipocytes have been shown to be an independent predictor of type 2 diabetes [18]. Further, a decrease in adipocyte cell size in those undergoing bariatric surgery has been shown to be more strongly associated with improved insulin sensitivity than a change in fat mass *per se* [19]. Despite a mean weight loss with apremilast, we did not observe any significant change in adipocyte size in this cohort. This may relate to the much more modest nature of weight loss with apremilast compared with that of bariatric surgery.

Despite previous studies demonstrating an association between weight loss and improved psoriatic disease activity [2, 3], there was no significant correlation between these parameters in the current study. This may relate to the more modest nature of weight loss with apremilast compared with stringent calorie reduction in these studies and suggests the principal mechanism of action of apremilast in PsA is likely through immune modulation rather than direct metabolic effects.

Overall, we did not observe any significant improvement in glycaemic status, GLP-1 or lipid parameters with apremilast. This contrasts with a recent study by Mazzilli *et al.* [20], who reported a reduction in glucose levels with apremilast. The discrepancy may relate to the nature of the study populations. The latter study included a significantly greater proportion of individuals with diabetes than the current study. Indeed, most of our participants were normoglycaemic at baseline, presenting a challenge in assessing glycaemic changes as glucose is tightly controlled. A post-hoc analysis of pooled data from 1808 participants from the ESTEEM, LIBERATE (NCT01690299) and PALACE (Efficacy and Safety Study of Apremilast to Treat Active Psoriatic Arthritis) trials, which included 163 participants taking anti-diabetic medications, revealed a modest improvement in HbA1c in those with an elevated baseline HbA1c ≥6.5% [7].

Fasting insulin concentration appeared to increase across study visits, together with an increase in HOMA-IR and corresponding decrease in HOMA insulin sensitivity. These changes appear contradictory to the previously noted beneficial glycaemic effects of PDE4 inhibition noted with other PDE4 inhibitors such as roflumilast [21] and are at odds with the overall weight loss and decreased disease activity seen in IMAPA participants. Whether this is a genuine effect of apremilast or a study effect remains to be ascertained and may warrant further study with a comparator placebo group. Despite these changes, there was no change in HbA1c, which is used for diagnostic and monitoring purposes in clinical practice.

In contrast to previous experimental models suggesting PDE4 inhibition may exert beneficial glycaemic and weight loss effects through enhanced GLP-1 release [8, 22], we did not observe any significant change in GLP-1 concentrations with apremilast over the 6 month period. This discrepancy with previous studies may result from inherent differences between animal and human models.

We did not observe any significant change in endothelial function or arterial stiffness with apremilast treatment. This may relate to the fact that our participants had reasonable baseline endothelial function, in contrast to a previous study that suggested PsA was associated with endothelial dysfunction [23].

The key strengths of this study lie in its extensive detailed longitudinal cardiometabolic phenotyping of patients with psoriatic disease undergoing treatment with apremilast through routine clinical care. We aimed to investigate the extent and nature of weight loss associated with apremilast through detailed MRI body composition analysis and adipose tissue biopsy. We also sought to examine whether PDE4 inhibition may exert potential metabolic benefits in psoriatic disease through enhanced GLP-1 activity using detailed analysis of pre- and post-prandial GLP-1 measurements during OGTT. Detailed disease activity phenotyping also allowed for clinical correlation of weight change with therapeutic response.

Study limitations included the lack of a comparator placebo group. Our findings should therefore be interpreted as exploratory and require validation in a larger, controlled study. The duration of follow-up to assess changes in body composition, adipocyte morphology and vascular function was also relatively short and may have limited the opportunity to detect a significant change in these.

In conclusion, following apremilast treatment we observed improvements in psoriatic disease activity and weight loss, primarily of abdominal subcutaneous fat. The clinical disease activity response with apremilast was independent of weight change, suggesting the main mechanism of action of apremilast in psoriatic disease is likely through direct immunological rather than metabolic effects.

## Supplementary Material

keab474_supplementary_data

## Data Availability

All relevant data are reported in the article. Additional details can be provided by the corresponding author upon reasonable request.
