# Supplementary material for: Effect of the phosphodiesterase 4 inhibitor apremilast on cardiometabolic outcomes in psoriatic disease—results of the Immune Metabolic Associations in Psoriatic Arthritis study
Source: Rheumatology (Oxford). 2021 Jun 7;61(3):1026–34. doi: 10.1093/rheumatology/keab474 (PMC8889283; doi:10.1093/rheumatology/keab474)
Supplement: keab474_supplementary_data [file keab474_supplementary_data.docx]

**Supplementary tables**

**Supplementary Table S1** Baseline disease activity parameters and change with apremilast treatment.

| Variable | Baseline  N=59 | Change at  Month 1  N=55 | P value | Change at  Month 3  N=53 | P value | Change at  Month 6  N=49 | P value |
| --- | --- | --- | --- | --- | --- | --- | --- |
| 66 Swollen Joint Count | 7 (3, 14) | -2.7 (-4.4, -1.1) | **0.001** | -2.5 (-4.2, -0.8) | **0.004** | -3.4 (-5.2, -1.7) | **<0.001** |
| 68 Tender Joint Count | 11 (5, 17) | -2.4 (-4.4, -0.4) | **0.021** | -1.8 (-3.8, 0.2) | 0.083 | -4.3 (-6.4, -2.2) | **<0.001** |
| DAS28-ESR | 4.4 (3.6, 5.4) | -0.3 (-0.6, -0.0) | **0.040** | -0.4 (-0.6, -0.1) | **0.015** | -0.5 (-0.8, -0.2) | **<0.001** |
| Patient global assessment | 59 (39, 76) | -7.7 ( -14.6, -0.8) | **0.030** | -8.7 (-15.7, -1.7) | **0.015** | -10.8 (-18.0, -3.6) | **0.003** |
| Physician global assessment | 57 (45, 72) | -18.6 (-24.8, -12.4) | **<0.001** | -22.5 (-28.7, -16.2) | **<0.001** | -25.1 (-31.6, -18.7) | **<0.001** |
| Pain VAS | 56 (33, 70) | -9.5 (-16.8, -2.2) | **0.011** | -11.3 (-18.6, -3.9) | **0.003** | -15.3 (-22.9, -7.7) | **<0.001** |
| LEI | 1 (0, 2) | -0.2 (-0.5, 0.1) | 0.211 | -0.5 (-0.9, -0.2) | **0.001** | -0.6 (-0.9, -0.2) | **0.001** |
| PASI | 3.8 (1.2, 9.2) | -2.4 (-3.7, -1.0) | **<0.001** | -2.6 (-4.0, -1.3) | **<0.001** | -1.9 (-3.3, -0.5) | **0.007** |
| HAQDI | 1 (0.38, 1.42) | -0.07 (-0.18, 0.03) | 0.181 | -0.06 (-0.16, 0.05) | 0.320 | -0.05 (-0.16, 0.07) | 0.411 |
| CRP (mg/dl) | 6 (3, 15) | -2.3 (-4.7, 0.2) | 0.070 | -3.5 (-6.0, -1.1) | **0.005** | -1.9 (-4.5, 0.6) | 0.136 |
| ESR (mm/hour) | 19 (6, 29) | 0.4 (-2.4, 3.3) | 0.764 | -2.1 (-5.0, 0.8) | 0.156 | -0.7 (-3.7, 2.2) | 0.625 |

Baseline values are median (IQR); mean change (95% CI) compared to baseline. Baseline disease activity data available for n=59. The values in bold are statistically significant, i.e. P <0.05.

**Supplementary Table S2** Correlation between percentage weight change with 6 months of apremilast treatment and disease activity parameters.

|  | Pearson’s correlation coefficient (r) | P value |
| --- | --- | --- |
| DAS28-ESR | -0.091 | 0.539 |
| 68 tender joint count | 0.065 | 0.657 |
| 66 swollen joint count | 0.005 | 0.975 |
| Patient global assessment | -0.379 | **0.007** |
| Physician global assessment | 0.061 | 0.679 |
| Pain VAS | -0.199 | 0.171 |
| LEI | -0.037 | 0.842 |
| Dactylitis count | -0.191 | 0.463 |
| HAQ-DI | -0.025 | 0.877 |
| PASI score | -0.015 | 0.921 |
| CRP | 0.027 | 0.855 |
| ESR | -0.002 | 0.988 |

N=49 with available data. The value in bold is statistically significant, i.e. P <0.05.

**Supplementary Table S3** Correlation between weight change with 6 months of apremilast treatment and fasting glucose, fasting insulin, and HOMA-IR.

|  | Pearson’s correlation coefficient (r) | P value |
| --- | --- | --- |
| Fasting glucose | -0.239 | 0.098 |
| Fasting insulin | -0.132 | 0.365 |
| HOMA-IR | -0.111 | 0.455 |

n=50 with available data

**Supplementary Table S4** Baseline vascular markers and change with apremilast treatment.

| Variable | Baseline | Change at  Month 1 | P value | Change at  Month 3 | P value | Change at  Month 6 | P value |
| --- | --- | --- | --- | --- | --- | --- | --- |
| Systolic BP (mmHg) | 132 (120, 146) | -1.1 (-4.9, 2.7) | 0.558 | -1.1 (-4.9, 2.8) | 0.584 | -4.1 (-8.1,  -0.1) | **0.042** |
| Diastolic BP (mmHg) | 78 (70, 84) | 2.6 (0.1, 5.1) | **0.040** | 1.8 (-0.7, 4.3) | 0.151 | -0.7 (-3.3, 1.9) | 0.591 |
| RHI† | 2.21 (1.82, 2.70) | - | - | -0.11 (-0.31, 0.08) | 0.240 | - | - |
| AI_@75_ (%) | 9.14 (1.51, 19.6) | - | - | 1.33 (-2.79, 5.44) | 0.528 | - | - |

Baseline values are median (IQR). Change represents mean difference (95% CI) compared to baseline. †RHI: reactive hyperaemia index, normal >1.67. EndoPAT data (RHI and Augmentation Index (AI@75)) available for n=38 at baseline, n=34 at month 3. Systolic and diastolic BP data available for n=59 at baseline, n=55 at month1, n=53 at month 3, and n=49 at month 6. The values in bold are statistically significant, i.e. P <0.05.
